# The American and Colonial Journals: Physiology

**Published:** 1839-01

**Authors:** 


### II. THE AMERICAN AND COLONIAL JOURNALS.

### PHYSIOLOGY.

### *On the Relation between the Respiratory and Circulating Functions.* By CHARLES HOOKER, M.D., of New Haven.

THE author sets out with stating that, from numerous careful observations, he considers the numerical relation between the number of respirations and the beats of the pulse to be as 1 to 4½ (except in early infancy); and this he regards as so constant that any considerable variation from it is a pretty sure indication of malformation or disease, provided that there is no mechanical impediment to the descent of the diaphragm, such as obesity, pregnancy, distention of the abdominal viscera, &c., which, by preventing the proper fulness of inspiration, will necessarily increase its frequency. From a disproportionate *increased* frequency of the respiratory movements, the general indication may be drawn that there is some impediment to the aeration of the blood; which may be owing to, 1, disorder of the lungs or air-passages; 2, some mechanical impediment to the motions of respiration; 3, imperfect function of the organic nerves of the lungs.

First, then, of disorder in the lungs and air-passages. In *pneumonia*, the disproportion is so marked as to be regarded by Dr. H. as almost a pathognomic sign; and, when the nature of the disease is ascertained, the relative frequency of the respirations affords a tolerable indication of the proportion of the lungs unfit for respiration. "In cases of extensive engorgement, it is not uncommon that the respiration is 45 in a minute, when the pulse does not exceed 90; the ratio becoming as 1 to 2. In extreme cases, the respiration becomes even 60 or 70; and in children I have occasionally noticed it 140 or 150. In less degrees of engorgement, the ratio is as 1 to 3, 3½, or 4. In the early stages of this disease, which so often advances in a *latent* form to a most injurious extent, the frequency of the respiration may advantageously direct attention to the state of the chest when the general symptoms are those of simple febrile disturbance. In the early stages of *phthisis*, when the only prominent general symptoms are a progressive debility and emaciation, "a disproportionate increased frequency of respiration affords a strong presumption of tubercular deposition. A simple general debility increases the frequency of respiration; but it occasions a proportionate increased frequency of the pulse, the ratio of 1 to 4½ is still preserved." In advanced stages of this disease, however, the proportion of blood in the system is so much diminished that a very small amount of aeration is required; and the frequency of the respiratory movements, therefore, does not bear any proportion to the quantity of the lungs unfit for service. The author notices *œdema* of the lungs as a very common cause of imperfect and therefore frequent respiration, and thinks that this state has been too little attended to. He also considers that there is a state which appears intermediate between proper inflammation and acute dropsy of the lungs, which might be termed œdematous inflammation, and which may lay claim to the title of a primary and idiopathic disease. This he states to have been the case in the epidemic influenza of 1831-2. Simple œdema he considers as almost universally present in general dropsical states, and to be very common in chlorosis and many cachectic diseases. We have ourselves noticed its occurrence in cases of the epidemic fever which may be characterized as severe synochus; and we have been accustomed to regard frequent and imperfect respiration, with extensive dulness on percussion, without cough or expectoration, as sufficient signs of its occurrence under those circumstances. We have always found the dulness diminish rapidly, and the respirations become slower, as the debility decreases; and in fatal cases, where death supervenes without any marked local affection, we have very commonly found an œdematous state of the lungs. It is unnecessary to enumerate the other diseases of the lungs and air-passages in which aeration will be necessarily imperfect, and the frequency of the respiratory movements increased.

Secondly, we may advert to the mechanical impediments which obstruct the respiratory movements, and thus increase their frequency. Of these, many are so obvious that they do not require to be enumerated; but under this head the author places also the "circumstances which render a full inspiration painful, as rheumatism or any inflammation of the intercostal or other muscles of respiration; or a like affection of the pleura, pericardium, heart, peritoneum, or any of the abdominal viscera." We do not think that this is exactly the place to class these causes of frequent respiration. Some of them (we would especially instance pericarditis and endocarditis) act, we are satisfied, on the respiratory movements, quite independently of the pain which full inspiration causes to the patient affected with them. We have scarcely ever noticed more frequent respiratory movements than in the advanced stages of acute pericarditis, when, adhesion of the two surfaces having takeu place, little pain was felt; and we are disposed to think that the state of the circulation in such cases has a more direct influence than Dr. H. seems to suppose.

Of the third cause of frequent respiration assigned by our author,—imperfect function of the ganglionic nerves of the lungs preventing the arterialization of the blood,—we must say that it seems to us purely hypothetical; and we can by no means regard the view which the author has taken of its operation as more than an ingenious explanation of certain known facts, which are capable of being accounted for without it. We certainly should not be inclined to allow it to influence our treatment, until much more evidence has been adduced in support of it than that brought forward by our author, who seems to regard the doctrine itself as sufficiently established to need no argument in its favour.

The author then goes on to consider the general pathological effects of imperfect aeration of the blood, and adopts the doctrines of Bichât on this subject, which, as we have heretofore observed, are quite incompatible with the experiments and observations of later authors, especially Drs. Kay and Williams. However, the *modus operandi* is in this instance a matter of secondary consequence, since all are agreed that the imperfect arterialization of the blood soon produces very deleterious effects on the system. Besides the causes already enumerated, imperfect aeration of the blood may occur from disordered function of the motor respiratory nerves. According to Dr. H. this is the case in *typhus* fever; and to it we are to attribute the relative slowness of the respiratory movements, which is always (according to him) remarkable in some stage of the fever, being commonly in the ratio of 1 to 5, or 1 to 6, with the pulse, and sometimes even as 1 to 7 or 8. This disordered function of the nerves he attributes to the "lesion of nervous function in the brain," forgetting, it would seem, that the brain, strictly so called, has nothing to do with the ordinary respiratory movements. That the general class of sympathetic or excited actions, of which the spinal cord is the channel, is greatly disturbed in this disease is familiar to every one; and, in its advanced stages, the action even of the muscles which guard the orifices of the alimentary canal is paralysed. In *delirium tremens*, also, Dr. H. regards infrequent respiration as an important symptom; and he considers it to be the cause of nightmare, being itself the result of pressure of the viscera on the nerves. Imperfect arterialization of the blood may be also due to disordered function of the sympathetic nerves, some degree of which is very common in typhoid complaints of all kinds, and especially manifests itself in malignant cholera and in the variety of asthma with puerile inspiration described by Laennec.

As there are few diseases in which the blood is *excessively* aerated, but, on the contrary, many in which there is deficient arterialization, our author considers the general therapeutic indication connected with the relation between the respiratory and circulating functions to be "to promote the arterialization of the blood, or, in other words, to remedy deficient respiration." He considers that "*stimulants*, which ordinarily operate to increase the action of the heart, without a corresponding increase of the respiration, should be withheld or given with extreme caution, when the blood is imperfectly arterialized;" but he allows that in some cases they are beneficial, and that their utility may be judged of by their effect on the respiratory function. On the other hand, he seeks for his remedies in the means which diminish the action of the heart and arteries, and thus tend to restore the balance and promote the arterialization of the blood; and in those which excite and invigorate the motor and organic nerves concerned in respiration. To the former class belong, of course, detraction of blood (so especially useful in cases where the pulse is *oppressed* by the influence of partial asphyxia), antimony, ipecacuanha, &c. and digitalis; which last the author states that he has employed to a large amount in the treatment of delirium tremens, and that, out of fifty cases of this disease, he has only lost four, in which there was a complication of disorders. The *modus operandi* of Dr. Graves's successful use of antimony combined with opium in the advanced stages of fever, he explains on his principles. The remedies which excite and invigorate the motor respiratory nerves are those termed *diffusible stimulants*, which Dr. H. regards as acting first on the brain, and then invigorating the system at large by their effect on the respiratory movements. The most novel part of his doctrines, however, consists in the recommendation of nitrate of silver as a medicine which has a particular influence in exciting and invigorating the arterializing (sympathetic) nerves of the lungs. In typhus fever and typhoid states of the system, constituting the malignant forms of scarlatina, erysipelas, &c., he places much reliance on this remedy, which he considers to have a marked influence also in delirium tremens, and combines with digitalis in the treatment of that disease. He gives it in doses of one eighth of a grain, repeated every hour, or once in two or three hours, according to the exigency of the symptoms. He illustrates its effects by a detailed and somewhat remarkable history of his own case of *erythema anatomicum;* in the relation of which, however, the influence of his preconceived views is sufficiently apparent. Calomel has also, he considers, a marked influence over the organic nerves of the lungs, and promotes the arterialization of the blood also by stimulating the other secretions. Those which are in any degree vicarious with respiration are, of course, to be promoted when the latter is deficient. Arterialization of the blood may also be assisted by ventilation, and by the removal of mechanical impediments to the respiration. One of the most common of these in low fevers is tympanitic distension of the alimentary canal, which Dr. H. states to be more relieved by nitrate of silver than by any other remedy.

[We have thus given a faithful account of Dr. Hooker's doctrines, in which there appears to us much that is sound as well as novel; but much, also, that requires confirmation by more extended experience. Unfortunately, Dr. H. leaves us almost entirely in the dark as to the extent of the data on which the results deduced by him are founded: we have, consequently, no means of estimating their value, without putting them again to the test of experience. We cannot but think also that, like many other men of ardent mind, he rides his hobby a little too far; but, perhaps, there are many who would not be induced to notice recommendations which are really of importance, if less urgently given.]

*Boston Med. and Surg. Journal. May and June,* 1838.

